# Preoperative Risk-assessment Score for Elective Total Aortic Arch Repair: AAORTA Risk Tool

**DOI:** 10.1055/a-2965-6246

**Published:** 2026-10-13

**Authors:** Michael Tyler Guinn, Travis J. Miles, Veronica A. Glover, Susan Y. Green, Lynna H. Nguyen, Ginger M. Etheridge, Lauren K. Barron, Anna H. Xue, Vicente Orozco-Sevilla, Subhasis Chatterjee, Marc R. Moon, Joseph S. Coselli

**Affiliations:** 1 Division of Cardiothoracic SurgeryMichael E. DeBakey Department of Surgery 3989 Baylor College of MedicineHoustonTexasUnited States; 2 Department of Bioengineering 3989 Rice UniversityHoustonTexasUnited States; 3 Office of Surgical ResearchMichael E. DeBakey Department of Surgery 3989 Baylor College of MedicineHoustonTexasUnited States; 4 The Texas Heart Institute at Baylor College of MedicineHoustonTexasUnited States; 5 Department of Cardiovascular Surgery 3989 CHI St Luke’s Health—Baylor St Luke’s Medical CenterHoustonTexasUnited States; 6 Cardiovascular Research Institute 3989 Baylor College of MedicineHoustonTexasUnited States

**Keywords:** aorta, total arch replacement, mortality risk score

## Abstract

**Objective:**

Total transverse aortic arch replacement is a complex operation performed to address aortic aneurysm with and without chronic aortic dissection. To predict 90-day survival after this procedure, we created a holistic tool for predicting postoperative mortality in patients who undergo total arch repair (TAR): the Aortic Arch Operation Risk Tool for Assessment (AAORTA) score.

**Methods:**

Univariate and multivariate analyses were performed on data from patients ( *N* =470; 243 with nondissecting aneurysm, 227 with chronic Type A dissection) who underwent TAR at a high-volume practice between April 2006 and November 2024. Regression analysis identified variables to be used in the AAORTA score. The predictive performance of the score was assessed with the area under the receiver operating characteristic curve (AUROCC), Brier score, and linear fitting.

**Results:**

Of 470 patients who underwent TAR, 65 (13.8%) died within 90 days of repair. The AAORTA score relied on five preoperative variables: heritable thoracic aortic disease, prior coronary artery bypass grafting (CABG), kidney disease, pulmonary disease (restrictive or obstructive), and no history of smoking. Previous CABG was the strongest predictor of 90-day mortality. The AAORTA score had an AUROCC of 0.784, a Brier score of 0.105, and a goodness-of-fit *R* ^2^ of 0.954.

**Conclusion:**

The AAORTA score can accurately stratify 90-day mortality risk in patients who undergo elective TAR, using only five variables easily acquired during standard preoperative evaluation. This score could be used to aid preoperative counseling with patients and families. Prospective multicenter validation will help establish its generalizability.

## Introduction

Mortality risk-estimation tools for patients who undergo surgery are practical and clinically relevant for surgeons and patients. 1​ ​2​ Such tools can stratify patient risk preoperatively and thus enrich dialogue with patients requiring surgical intervention about possible clinical courses that lie ahead. Many scores rely on preoperative characteristics and therefore can be calculated before surgery, allowing risk stratification and, possibly, optimization of patient factors before the planned procedure. 3​ ​4​ For example, a frailty score for aortic surgery patients was developed in 113 such patients; however, no non-frailty variables were assessed, and no validation was done. 5​ Such tools could improve risk prediction in aortic surgery, but no comprehensive score related to thoracic aortic surgery has been created to date.

Aortic surgery, a crucial aspect of modern cardiac surgery, has continued to evolve since the first description by De Bakey and colleagues. 6​ This discipline often deals with aortic dissection and thoracic aortic aneurysm. Each of these conditions can be repaired by total arch replacement (TAR), an extensive but potentially life-saving procedure 7​ ​8​ that often involves specialized techniques for cannulation, cerebral perfusion, and brachiocephalic vessel reimplantation. 9​ 10​ ​11​ Additionally, analysis of thoracic endovascular aortic repair versus open repair has shown adequate outcomes for both procedures. Whereas previous aortic studies have investigated how risk factors are associated with mortality in patients who undergo TAR, no study has produced a comprehensive risk score that can predict mortality. 12​ ​13​ Building such a score for patients who require TAR could give clinicians a tool to stratify risk for these patients.

To address this lack of risk-assessment tools for aortic surgery, we created a comprehensive risk score to estimate 90-day postoperative mortality risk for patients who undergo elective TAR. This score, named the Aortic Arch Operation Risk Tool for Assessment (AAORTA) score, uses preoperative variables exclusively to predict outcomes of elective aortic arch replacement. The purpose of this study was to develop and validate this risk score.

## Materials and Methods

### Data Collection and Study Population

A prospectively maintained database of complex aortic surgical procedures was queried for data from April 2006 to November 2024. Baylor College of Medicine’s institutional review board (IRB) approved our clinical research protocol (#18095) in 2006. Informed consent was obtained from all patients who underwent repairs after this approval. Written consent was secured using the IRB-approved form. For underage patients, consent was obtained from the parent or guardian. For patients who were incapacitated or died before consent could be obtained and who had no available family members to consent on their behalf, the IRB granted a waiver of consent. For patients treated before 2006, data were collected retrospectively under an IRB-approved waiver. The year 2006 was chosen as the beginning of the study period because that year marked the start of prospective data collection, developments in clinical approach such as antegrade cerebral perfusion strategies and warmer absolute temperatures in hypothermic circulatory arrest, and the standardization of clinical practices at our institution, as well as to create a contemporary dataset.

Patients who had elective TAR via a median sternotomy, were without prior arch surgery, were at least 18 years old, and did not have an acute or subacute dissection were included in the study. Thus, we excluded patients with hemiarch or partial arch surgery and those with acute or subacute dissection; additionally, we removed a small number of outliers, such as patients with simultaneous open arch and open descending thoracic/thoracoabdominal aortic replacement performed via alternate incision and patients who underwent TAR for reasons other than degenerative aneurysm or chronic dissection (e.g., aortic tumor, infection). Key variables are defined in **Supplementary Table S1** (available in the online version only). Survival information was obtained 30 days postoperatively and yearly thereafter by clinic visits, telephone interviews, or searching the Social Security Death Index. Patient data were deidentified.

### Aortic Arch Operation Risk Tool for Assessment (AAORTA) Score Calculation

Patients were randomly assigned to training (80%, *n* =376) and testing sets (20%, *n* =94) to prevent selection bias and ensure reproducibility of the model. Missing data, which were rare, were imputed by the median imputation method for the entire dataset. Although the training and testing sets were partitioned randomly, there was a small but statistically significant difference between sets in the prevalence of prior aortic valve replacement (AVR) or aortic root replacement; this difference represents a stochastic distribution artifact inherent to random sampling in smaller cohorts, rather than systemic bias. For the final cohort, in the training dataset, 38 preoperative variables ( Table 1 ) were analyzed univariately for any association, positive or negative, with mortality at 90 days. The colinear variables were then removed, and clinically relevant variables were analyzed for any univariate association with 90-day death. To avoid premature exclusion of clinically important variables, those with *p* -value≤0.2 were included in multivariate analysis. 14​ Backwards stepwise feature selection was then used to identify predictors of mortality. Variables from this analysis were used in the AAORTA score, which was computed from these variables’ beta coefficients (used to determine the odds ratio) in the training set. The smallest beta coefficient from backward stepwise feature selection was used to normalize all coefficients, which yielded the point value that each variable would contribute to the AAORTA score.

**Table:** **Table 1** Preoperative factors assessed in Aortic Arch Operation Risk Tool for Assessment (AAORTA) score

Patient age>60 y	Body surface area≥2 m ^2^
Gender (male/female)	BMI<18 kg/m ^2^
Heritable thoracic aortic disease	BMI 18–25 kg/m ^2^
Aneurysm without dissection	BMI 25–30 kg/m ^2^
Chronic dissection	BMI 30–35 kg/m ^2^
More than 1 dissection	BMI>35 kg/m ^2^
Hypertension	Pulmonary disease
Hyperlipidemia	Currently using tobacco
Diabetes	Never used tobacco
History of arrhythmia	Formerly used tobacco
Coronary artery disease	Obstructive sleep apnea
Prior CABG	Gastrointestinal disease
Previous myocardial infarction	Peripheral vascular disease
NYHA class	Ejection fraction mildly reduced (41–49%)
Cerebrovascular disease	Ejection fraction moderately reduced (30–40%)
Transient ischemic attack	Ejection fraction severely reduced (<30%)
Kidney disease (general)	Prior AVR or aortic root replacement
CKD (eGFR<60 mL/min/1.73 m ^2^ )	Prior ascending aorta replacement
Preoperative creatinine<2 mg/dL	Redo sternotomy (operative variable)

Abbreviations: AVR, aortic valve replacement; BMI, body mass index; CABG, coronary artery bypass grafting; CKD, chronic kidney disease; eGFR, estimated glomerular filtration rate; NYHA, New York Heart Association.

Note: Redo sternotomy was treated as a preoperative variable because all redo operations were planned in advance.

The area under the receiver operating characteristic curve (AUROCC) was used to assess the accuracy of the scoring system in the testing set. The AAORTA score was binned into three categories of risk—high, medium, and low—to assess its predictive value for mortality and was tested with the log-rank test on a Kaplan–Meier plot for the entire dataset.

### Statistical Analysis

Data analysis was performed in R Studio (version 2024.09.1+394) using standard statistical packages. Comparative analyses were performed using the Fisher exact test or the Wilcoxon rank-sum test, as appropriate. For the AAORTA score, patient death within 90 days was the primary outcome used in Kaplan–Meier analysis with comparison by the log-rank test. Death within 90 days was the dependent variable, while patients’ preoperative variables were the independent variables that were investigated by univariate and multivariate analyses. The Wald test was used to assess numerical variables (including categorical variables converted to numerical variables). Lastly, Brier score and goodness-of-fit *R* ^2^ value were used to compare predicted mortality (determined from AAORTA scores) with actual 90-day mortality.

## Results

### Study Cohort Characteristics

Of 3,353 patients who underwent open aortic arch repair at a high-volume practice, 817 had TAR. Patients were excluded who had prior arch surgery ( *n* =17), died intraoperatively ( *n* =9), were younger than 18 years old ( *n* =2), underwent surgery before 2006 ( *n* =242), had an acute or subacute dissection ( *n* =22), required urgent or emergency surgery ( *n* =46), underwent antegrade thoracic endovascular aortic repair (TEVAR; *n* =7), had an extended repair that included the descending thoracic aorta ( *n* =1), or had an aortic tumor ( *n* =1). Of the 470 patients (66.6% male, median age 64 years) remaining in the dataset, 243 (51.7%) had degenerative aneurysm without dissection, and 227 (48.3%) had chronic dissection. Of the patients, 80% ( *n* =376) were allocated to the training dataset; the other 20% ( *n* =94) were allocated to the validation dataset ( Fig. 1 ). In the overall cohort, most patients had hypertension (90.0%) and were current or former smokers (59.4%), and median body mass index was 27.5 [24, 31] kg/m ^2^ ( Table 2 ). A minority of patients had heritable thoracic aortic disease (HTAD; 23.4%). Notably, 56.2% of patients underwent redo sternotomy as part of elective TAR ( Table 3 ).

**Fig. 1:**
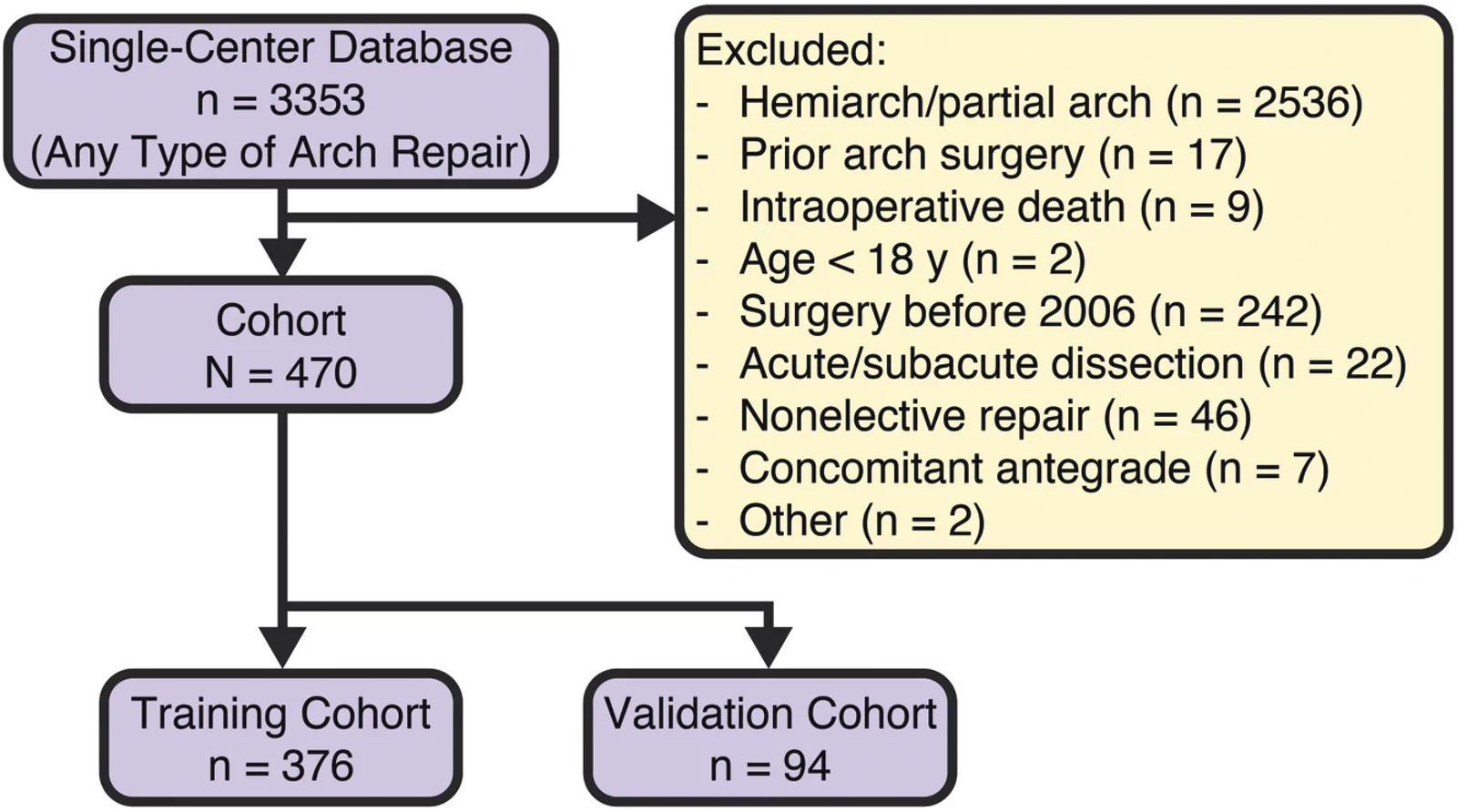
Inclusion criteria for the Aortic Arch Operation Risk Tool for Assessment (AAORTA) score study. Regarding the other exclusions, they included a patient with concomitant repair of the descending thoracic aorta and a patient with an aortic tumor. DTA, descending thoracic aorta; TAAA, thoracoabdominal aortic aneurysm; TAR, total arch replacement; TEVAR, thoracic endovascular aortic repair.

**Table:** **Table 2** Preoperative characteristics of 470 patients who underwent total arch replacement

Characteristics	Overall; *N* =470	Test (20%); *n* =94	Train (80%); *n* =376	*p* -Value
Age (years)	64 [54, 71]	64 [55, 70]	64 [54, 71]	0.6 ^a^
Age>60 years	285 (60.6)	59 (62.8)	226 (60.1)	0.723
Male	313 (66.6)	60 (63.8)	253 (67.3)	0.542
Heritable thoracic aortic disease	110 (23.4)	20 (21.3)	90 (23.9)	0.683
Aneurysm (no dissection)	243 (51.7)	45 (47.9)	198 (52.7)	0.421
Chronic aortic dissection	227 (48.3)	49 (52.1)	178 (47.3)	0.421
Multiple dissections	15 (3.2)	4 (4.3)	11 (2.9)	0.514
Body surface area≥2 m ^2^	251 (53.4)	51 (54.3)	200 (53.2)	0.908
Max. proximal diameter (cm)	5.6 [5.0, 6.3]	5.4 [4.6, 6.2]	5.6 [5.0, 6.3]	0.607 ^a^
Hypertension	423 (90.0)	88 (93.6)	335 (89.1)	0.249
Hyperlipidemia	225 (47.9)	45 (47.9)	180 (47.9)	>0.999
Diabetes mellitus	34 (7.2)	7 (7.4)	27 (7.2)	>0.999
History of arrhythmia	96 (20.4)	22 (23.4)	74 (19.7)	0.474
Coronary artery disease	143 (30.4)	23 (24.5)	120 (31.9)	0.170
Prior CABG	27 (5.7)	3 (3.2)	24 (6.4)	0.323
Prior myocardial infarction	45 (9.6)	4 (4.3)	41 (10.9)	0.051
Ejection fraction (%)	58 [55, 63]	60 [55, 63]	57 [55, 63]	0.139 ^a^
Mildly reduced (41–49%)	79 (16.8)	13 (13.8)	66 (17.6)	0.443
Moderately reduced (31–40%)	25 (5.3)	3 (3.2)	22 (5.9)	0.441
Severely reduced (≤30%)	1 (0.2)	0	1 (0.3)	>0.999
Heart failure (NYHA Class 3 or 4)	110 (23.4)	25 (26.6)	85 (22.6)	0.416
Cerebrovascular disease	70 (14.9)	12 (12.8)	58 (15.4)	0.628
Transient ischemic attack	18 (3.8)	4 (4.3)	14 (3.7)	0.767
Kidney disease (general)	143 (30.4)	31 (33.0)	112 (29.8)	0.534
CKD (eGFR<60 mL/min/1.73 m ^2^ )	113 (24.0)	26 (27.7)	87 (23.1)	0.349
Preop creatinine<2 mg/dL	464 (98.7)	92 (97.9)	372 (98.9)	0.345
BMI (kg/m ^2^ )	27.5 [24, 31]	28 [24, 31]	27.5 [25, 31]	0.837 ^a^
Underweight (BMI<18.5)	13 (2.8)	2 (2.1)	11 (2.9)	0.864
Normal weight (18.5≤BMI<25)	127 (27.0)	29 (30.9)	98 (26.1)	0.365
Overweight (25≤BMI<30)	197 (41.9)	36 (38.3)	161 (42.8)	0.483
Obese Class I (30≤BMI<35)	92 (19.6)	18 (19.1)	74 (19.7)	>0.999
Obese Class II or III (BMI≥35)	41 (8.7)	9 (9.6)	32 (8.5)	0.688
Pulmonary disease	174 (37.0)	35 (37.2)	139 (37.0)	>0.999
Current tobacco use	54 (11.5)	15 (16.0)	39 (10.4)	0.147
Never smoked	190 (40.4)	39 (41.5)	151 (40.2)	0.815
Former smoker	225 (47.9)	40 (42.6)	185 (49.2)	0.299
Obstructive sleep apnea	70 (14.9)	18 (19.1)	52 (13.8)	0.198
Gastrointestinal disease	164 (34.9)	29 (30.9)	135 (35.9)	0.398
Peripheral vascular disease	75 (16.0)	16 (17.0)	59 (15.7)	0.754
Prior AVR or root replacement	89 (18.9)	10 (10.6)	79 (21.0)	0.026
Prior ascending replacement	195 (41.5)	40 (42.6)	155 (41.2)	0.816

Abbreviations: AVR, aortic valve replacement; BMI, body mass index; CABG, coronary artery bypass grafting; CKD, chronic kidney disease; EF, ejection fraction; eGFR, estimated glomerular filtration rate; NYHA, New York Heart Association.

Note: *p* -Values show Fisher exact test unless otherwise indicated.

Data are *n* (%) or median (quartile 1, quartile 3).

^a^ Wilcoxon rank-sum test. Although the training and testing sets were partitioned randomly, there was a small but statistically significant difference between sets in the prevalence of prior AVR or aortic root replacement; this difference represents a stochastic distribution artifact inherent to random sampling in smaller cohorts, rather than systemic bias.

**Table:** **Table 3** Operative details of 470 patients who underwent total arch replacement

Characteristics	Overall; *n* =470	Test (20%); *n* =94	Train (80%); *n* =376	*p* -Value
Redo sternotomy	264 (56.2)	50 (53.2)	214 (56.9)	0.10
Distal anastomosis (select)				
Reverse elephant trunk	14 (3.0)	4 (4.3)	10 (2.7)	0.5
Classic “soft” elephant trunk	149 (31.7)	33 (35.1)	116 (30.9)	0.38
Frozen elephant trunk	169 (36.0)	32 (34.0)	137 (36.4)	0.7
Proximal procedures				
Aortic root replacement	57 (12.1)	14 (14.9)	45 (12.0)	0.5
Aortic valve replacement (non-root)	81 (17.2)	24 (25.5)	57 (15.2)	0.022
Concomitant procedures				
Coronary artery bypass grafting	57 (12.1)	15 (16.0)	42 (11.2)	0.2

Note: Data are *n* (%).

### Univariate Analysis and Backward Stepwise Feature Selection

Univariate analysis was performed on 38 variables ( Table 1 ) in the training dataset. The 11 variables that significantly predicted 90-day mortality on univariate analysis then underwent backward stepwise feature selection, which identified five of these variables as positive or negative independent predictors of 90-day postoperative mortality: HTAD, prior coronary artery bypass grafting (CABG) surgery, kidney disease, pulmonary disease, and no history of smoking ( **Supplementary Table S2** , available in the online version only). These factors were used to compute the AAORTA score in both the training and testing datasets. HTAD and no history of smoking were negative predictors of 90-day mortality, and prior CABG was the strongest positive predictor.

### Aortic Arch Operation Risk Tool for Assessment (AAORTA) Score

The AAORTA score was generated and displayed as an AUROCC graph ( Fig. 2 ) for the testing dataset. When used to predict 90-day mortality in the testing dataset, the score’s AUROCC was 0.784 (95% confidence interval [CI]: 0.651–0.917). The score’s sensitivity was 76.9% and its specificity was 66.7%. The logistic regression coefficients, odds ratios with 95% CIs, and allocation of integer score values (i.e., points) for each risk factor in the training set are provided in **Supplementary Table S2** (available in the online version only). An online application ( https://tar-aaorta-score.streamlit.app/ , **Supplementary Fig. S1** , available in the online version only) was developed to calculate the AAORTA score.

**Fig. 2:**
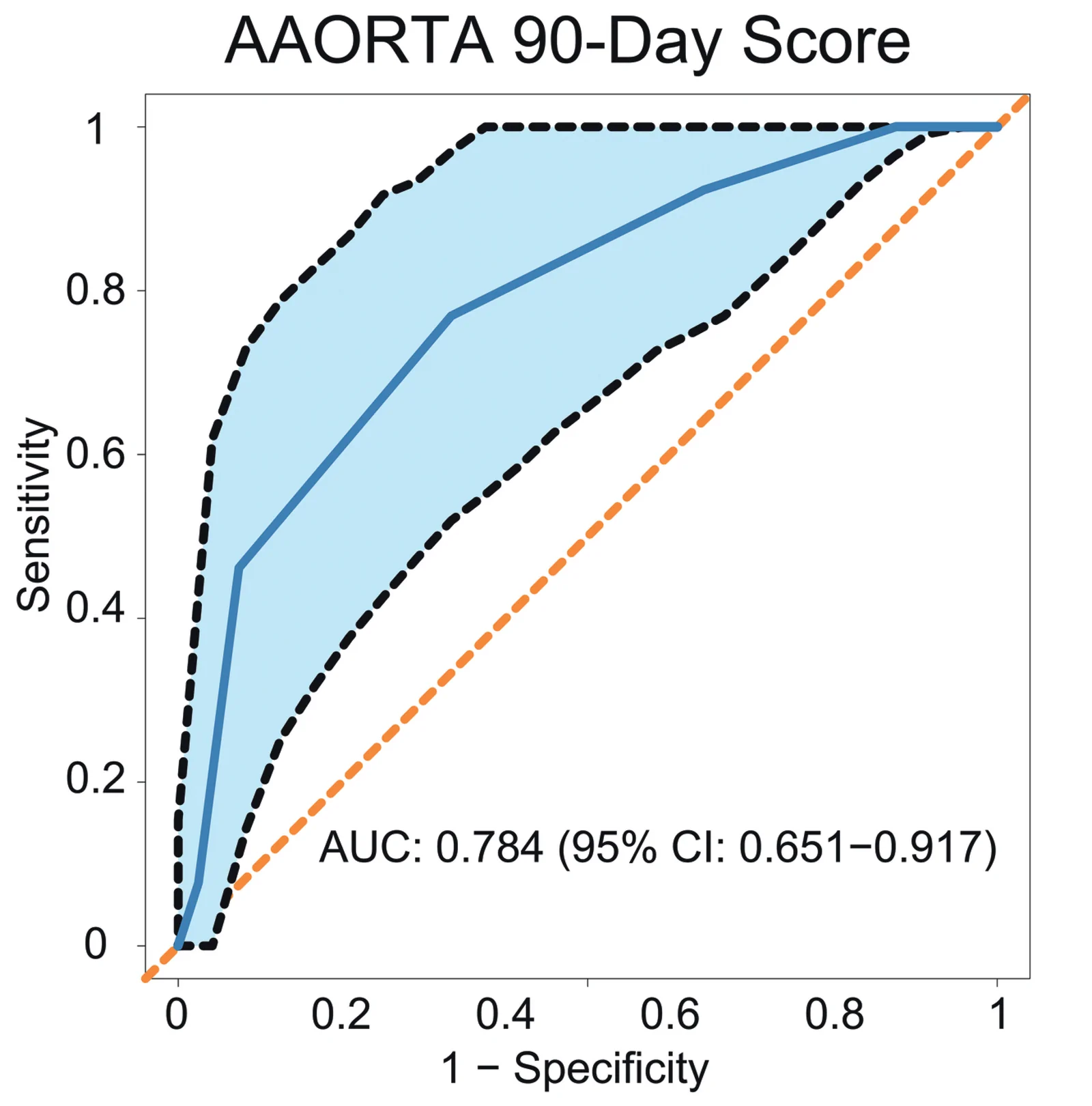
Area under the receiver operating characteristic curve (AUROCC) for Aortic Arch Operation Risk Tool for Assessment (AAORTA) score at 90 days after total arch replacement surgery in the testing set. The AAORTA score is computed from preoperative factors. The blue line is the mean AUROCC, while the dotted black lines are the lower and upper 95% confidence interval bounds. The dotted red line is the line of no-discrimination.

In the full dataset, the AAORTA score ranged from−2 to 4; higher scores were associated with higher risk of mortality ( Fig. 3 ). The median AAORTA score for the cohort was 0 (quartile 1, quartile 3:−1, 1). Patients were divided into three risk categories according to AAORTA score—low risk (≤0), medium risk (1), and high risk (>1) ( **Supplementary Table S3** , available in the online version only)—and their survival was assessed with Kaplan–Meier curves, which were significantly different from one another ( Fig. 3 ).

**Fig. 3:**
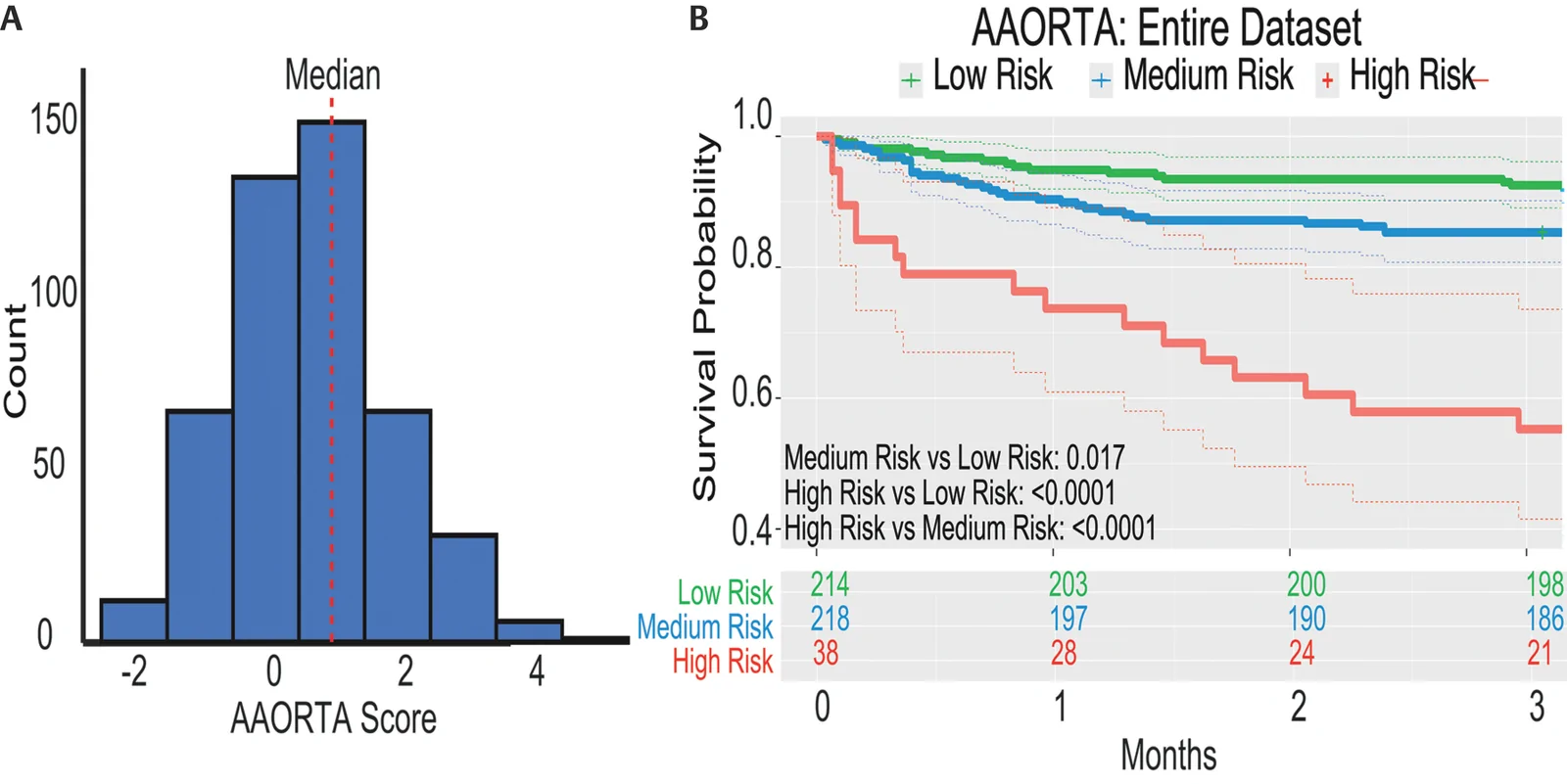
Score distribution and Kaplan–Meier (KM) curves in the full dataset. ( **A** ) Aortic Arch Operation Risk Tool for Assessment (AAORTA) score distribution among the 470 patients. The score ranged from−2 to 4, and the red dotted line represents the median score of the dataset. ( **B** ) KM curves for AAORTA score at 90 days after total arch replacement surgery in the full cohort. AAORTA score was binned into three groups: low risk, medium risk, and high risk. *p* -Values for differences between groups were generated by log-rank tests. The number of surviving patients in each risk category is displayed under the KM graph across time. Dotted lines represent 95% confidence intervals.

Lastly, we used logistic regression to assess the AAORTA score’s accuracy in predicting 90-day mortality in the testing dataset. Patients were grouped into the same low-risk, medium-risk, and high-risk groups. The logistic regression model produced expected mortality rates based on the AAORTA score that were close to the observed mortality rates in the validation cohort ( Fig. 4 ). When we tested the reliability of this model, we found a high goodness-of-fit *R* ^2^ value of 0.954, showing that the predicted and observed mortalities were highly aligned in the testing dataset. Furthermore, the Brier score was low (0.105), indicating strong predictive power. The 90-day survival rate predicted by logistic regression from the AAORTA score was 91.5% (95% CI: 87.4–94.3%) in the low-risk group, 82.1% (95% CI: 77.0–86.2%) in the medium-risk group, and 69.8% (95% CI: 58.4–79.4%) in the high-risk group. The actual survival rate was 94.7% in the low-risk group, 84% in the medium-risk group, and 50% in the high-risk group. The overall 90-day death rate was 13.8%. In comparison, the overall 30-day death rate was 8.9%, indicating that not all operative risk was fully captured within this timeframe.

**Fig. 4:**
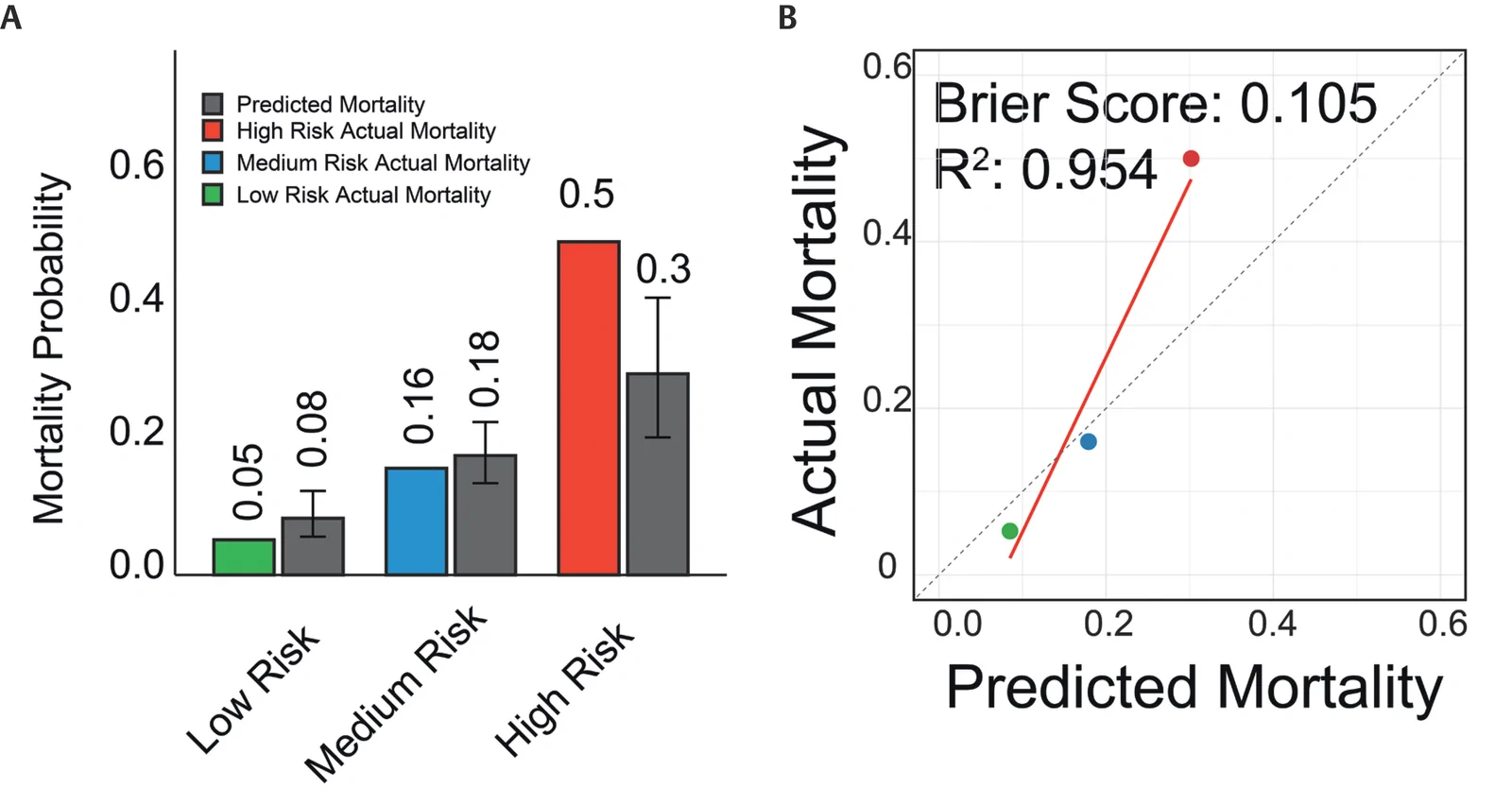
Mortality risk at 90 days after total arch replacement surgery in the testing set. ( **A** ) Predicted (gray bars) and actual mortality (green, blue, and red bars) of patients stratified into low-risk, medium-risk, and high-risk groups by using the Aortic Arch Operation Risk Tool for Assessment (AAORTA) score. Predicted mortality was derived from the logistic regression analysis. Error bars represent 95% confidence interval. Values above bars represent probability or mortality. ( **B** ) Calibration plot of predicted versus observed mortality from panel **A** . The solid red line is the best fit line between the groups, and the dotted black line represents a perfect correlation between prediction and observation.

## Discussion

This study showed that 90-day mortality after TAR can be predicted from a few preoperative variables. The ability to predict postoperative mortality is useful for all cardiothoracic surgical patients and especially for patients with chronic aortic dissection or thoracic aortic aneurysm requiring TAR, which can be a high-risk procedure. However, there are no established, user-friendly risk scores to predict aortic surgery–related mortality. Studies have associated certain factors like past or current smoking, history of cardiac disease, and heart failure with postoperative mortality. 15​ Yet no one has incorporated all these factors into an easily calculable score for risk prediction.

To address this gap, we created the comprehensive AAORTA score, a preoperative risk tool that estimates TAR patients’ risk of death within 90 days of surgery. Among 38 preoperative variables, we identified 5 independent predictors (3 positive, 2 negative) of 90-day mortality to use in the AAORTA score. The strongest predictor of mortality was previous CABG. Never having smoked was associated with lower mortality risk, allowing clinicians to possibly classify these patients as low risk for elective TAR surgery. The AAORTA score had an AUROCC of 0.784 (95% CI: 0.651–0.917), indicating strong predictive power. This result was corroborated by the predicted mortality matching observed mortality with high accuracy as indicated by a low Brier score (0.105) and a high *R* ^2^ value (0.954). Interestingly, HTAD was found to be protective, perhaps because patients with HTAD undergo more routine screening, are younger, and have fewer comorbidities, leading to better survival. Other variables like reduced ejection fraction or history of arrhythmias were not found to be associated with mortality; however, this may be because our cohort did not include enough patients with moderate or severely reduced ejection fraction or arrhythmia history for any such association to be statistically significant. Lastly, to effectively implement the AAORTA score in the real world, surgeons need an easy calculation method. For this, we developed an online application ( https://tar-aaorta-score.streamlit.app/ , **Supplementary Fig. S1** (available in the online version only)) to enable clinicians to rapidly assess elective TAR risk by entering five patient parameters. With external validation, adding these and other variables may further improve the AAORTA score.

The limitations of this study include being a single-center study and the limited number of clinical variables captured in our database. Additionally, whereas the model performed well as evidenced by its high *R* ^2^ value, the high-risk group in the model underestimated the actual mortality, highlighting a future opportunity for model improvement. Although the score performed well on internal validation with training and testing datasets, it also requires external validation. The Society of Thoracic Surgeons (STS) database is an ideal dataset for external validation of the AAORTA score 16​ because it includes several variables used in the score, including pulmonary disease, tobacco use, and previous CABG. The STS database also captures creatinine levels, from which renal disease can be identified indirectly. Lastly, HTAD is not captured in the STS dataset but is an important variable in the score. Although the STS database is not perfectly aligned with our exploratory model, a future study using STS data after HTAD has been incorporated would have sufficient commonality with our dataset to assess the generalizability of our findings.

Also, while the five variables included in the score produced a highly accurate AUROCC, there are probably other important factors that should be included in future versions of the AAORTA score to boost its utility. Additionally, whereas this study focused on elective TAR, other regional centers may perform TAR with different surgical techniques and different protocols, including those for temperature regulation and organ protection. Some centers may predominantly focus on urgent cases with acute dissection, limiting the application of the AAORTA score to nonelective surgical patients.

Despite these limitations, this study generated a novel scoring method for estimating risk in patients who undergo TAR. This risk score can be easily calculated at the bedside from a few variables. Also, as analytic tools continue to evolve, in addition to logistic regression, machine learning methods may further improve the performance of this score by revealing new crucial variables that are not intuitive clinically.

It is possible that the AAORTA score could be used in the future to identify patients at abnormally high surgical risk who might become good candidates for endovascular repair as the tools for it evolve. Prospective randomized trials will be needed to determine whether patients requiring TAR would benefit from such a change in clinical practice. At the minimum, the AAORTA lays the groundwork for identifying high-risk patients who have approximately 50% mortality risk at 90 days, who in the future may be included in such a randomized trial. Currently, the AAORTA score is well positioned to be used by clinicians to stratify risk in patients with plans to undergo TAR at high-volume aortic centers. Additionally, whenever possible, the patient’s preoperative risk factors can be addressed before operation if the surgery is not immediately necessary. In summary, the AAORTA score can be easily used to stratify patients into groups with low, medium, and high mortality risk to help guide clinical decision-making.

## C﻿onclusion﻿

The AAORTA score stratifies 90-day mortality risk in patients undergoing elective total arch replacement by using five readily available preoperative variables: heritable thoracic aortic disease, prior coronary artery bypass grafting (CABG), kidney disease, pulmonary disease, and no history of smoking. The score showed good discrimination (AUROCC 0.784) and separated patients into low-, medium-, and high-risk groups with close agreement between predicted and observed mortality. External, multicenter validation is needed before broader clinical use.

## Data Availability

The data underlying this article are available in the article and its online supplementary material. The raw data cannot be shared publicly to protect the privacy of the individuals who participated in the study.

## Supplementary Material

Supplementary Material Zusatzmaterial
