# Supplementary material for: Preoperative Risk-assessment Score for Elective Total Aortic Arch Repair: AAORTA Risk Tool
Source: Aorta (Stamford). 2026 Oct 13;14:a29656246. doi: 10.1055/a-2965-6246 (PMC13662675; doi:10.1055/a-2965-6246)

Supplementary Table 1 Standardized definitions of preoperative predictors used to compute AAORTA score

Predictor	Definition
Heritable thoracic aortic disease	Documentation of a connective tissue disorder or thoracic aortic repair at age ≤50 years
Hyperlipidemia	Documentation of a diagnosis hyperlipidemia, based on longitudinal lipid measurements; commonly, this information is captured in the history & physical evaluation
Prior coronary artery bypass grafting	Documentation of prior surgery; does not capture the number or source of prior grafts
Kidney disease (general)	Preoperative renal insufficiency (dysfunction) with serum creatinine >3.0 mg/dL, renal failure necessitating dialysis, prior renal transplant, atrophic kidney, partial or whole previous nephrectomy, polycystic kidney disease, nephritis, nephrolithiasis, and other specified renal diseases
Pulmonary disease	Included asthma, chronic obstructive pulmonary disease, obstructive sleep apnea, prior lung transplant, tuberculosis, pneumothorax, and other documented chronic pulmonary conditions
Never used tobacco	Patient confirmed they had never used tobacco.

Abbreviation: AAORTA, Aortic Arch Operation Risk Tool for Assessment.

Supplementary Table 2 Preoperative predictors used to compute AAORTA score

Predictor	Count, n (%)	Beta Coefficient	Odds Ratio (95% CI)	Points
HTAD	90 (23.9)	−0.55	0.57 (0.25-1.31)	−1
Prior CABG	24 (6.4)	1.08	2.95 (1.05-8.25)	2
Kidney disease	112 (29.8)	0.58	1.78 (0.96-3.32)	1
Pulmonary disease	139 (37.0)	0.69	2.00 (1.09-3.68)	1
Never used tobacco	151 (40.2)	−0.72	0.49 (0.24-1.00)	−1

Beta coefficients from the multivariate analysis model in the training set (n=376) were used to compute odds ratios and AAORTA scores. Abbreviations: AAORTA, Aortic Arch Operation Risk Tool for Assessment; CABG, coronary artery bypass grafting; CI, confidence interval; HTAD, heritable thoracic aortic disease.

Supplementary Table 3 Risk groups generated from AAORTA score

Risk group	Point range	Percentage of patients	*p*-Value
Low risk	≤0	61.5	Reference
Medium risk	1	26.2	0.035
High risk	>1	12.3	<0.0001

p-Value is based on the log-rank test between the reference ‘low-risk’ score group.

Abbreviations: AAORTA, Aortic Arch Operation Risk Tool for Assessment.

Supplementary Fig. 1. Screenshots from the Web-based AAORTA score calculator, which can be accessed here: https://tar-aaorta-score.streamlit.app/

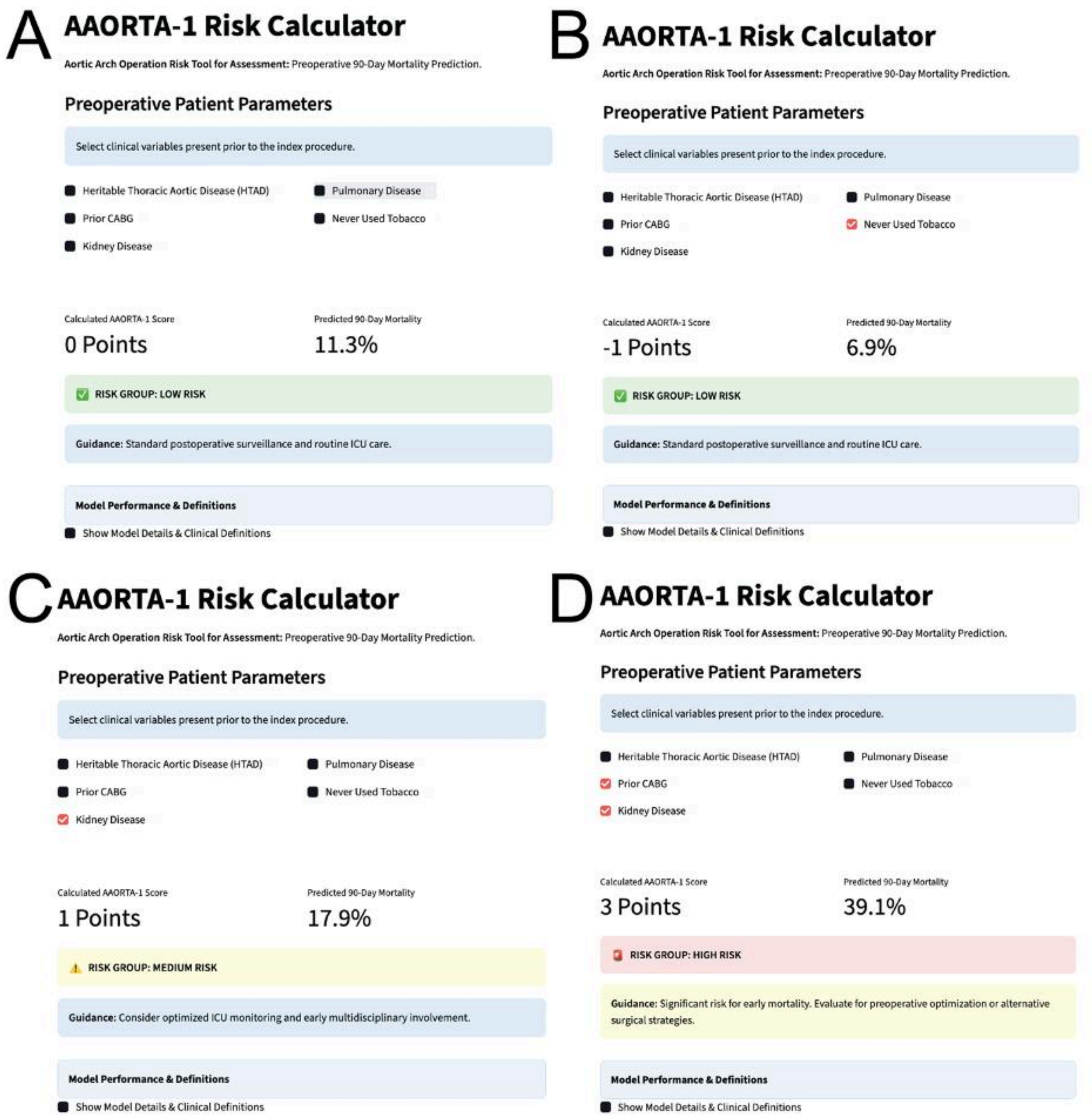

Supplement: Supplementary file 1 [file 10-1055-a-2965-6246_29736867.pdf]
